# Source and variation of the amazing live Sea-Monkey microbiome

**DOI:** 10.1371/journal.pone.0308763

**Published:** 2024-08-12

**Authors:** Corey C. Holt, Javier del Campo, Patrick J. Keeling

**Affiliations:** 1 Department of Botany, University of British Columbia, Vancouver, British Columbia, Canada; 2 Hakai Institute, Heriot Bay, British Columbia, Canada; 3 Institut de Biologia Evolutiva (CSIC-Universitat Pompeu Fabra), Barcelona, Catalonia, Spain; Sathyabama Institute of Science and Technology, INDIA

## Abstract

An embryonic diapause in unfavourable conditions has allowed brine shrimp to thrive in hypersaline environments and, unexpectedly, mail-order sachets and small, novelty tanks. Marketed as Sea-Monkeys®, each kit involves a 3-step process to generate adult *Artemia* within a matter of weeks. Whether these kits also allow for the maintenance of a host-associated microbiome is unclear. Therefore, comparing five replicate tanks under the same culture conditions, we sequenced the 16S ribosomal small subunit (SSU) gene to analyse bacterial community compositions in adults, their surrounding tank water, and their feed. Adult Sea-Monkeys® harboured a bacterial microbiome that was clearly distinguishable from the tank water and food. Furthermore, individual tanks had a notable effect on fine-scale microbiome variation. Several Sea-Monkey bacterial variants appeared absent in environmental samples and included genera (*Leucobacter* and *Microbacterium*) known to confer desiccation resistance in other hosts. Although Sea-Monkeys® taxonomy is unclear, phylogenetic inference of the cytochrome c oxidase I (COXI) gene from the host animal suggests Sea-Monkeys® belong to the *Artemia franciscana* ‘superspecies’. Overall, Sea-Monkeys® kits appear to be a convenient and scalable mesocosm for the study of host-microbiome interactions and could serve as a useful tool for future invertebrate microbiome research, outreach, and education.

## Introduction

Brine shrimp (*Artemia* spp.) are small, filter-feeding crustaceans that are typically found in hypersaline lakes [1]. Their success and subsequent proliferation in nature, as evidenced by their widespread geographic distribution, is in part due to the absence of most predators in the harsh conditions in which they thrive [1]. *Artemia* are, however, an important food source in personal and commercial aquaria; 3500–4000 tonnes of *Artemia* eggs are produced each year for feed [2].

Key to their success in both the commercial sector and their natural habitat is their developmental flexibility. *Artemia* can reproduce either ovoviviparously (i.e., offspring develop internally and are released after hatching) or oviparously (i.e., offspring develop and hatch externally), depending on environmental conditions [1]. In poor conditions, oviparous gastrulae are encysted and enter diapause–a technique, often referred to as cryptobiosis or anhydrobiosis, which involves the animal or, in this case, its embryos, entering a dormant, metabolic state within which it can resist long bouts of desiccation, low temperature, and/or anoxia [1]. Several animals, including tardigrades [3], rotifers [4], and nematodes [5] are also known to use some form of cryptobiosis. This process in *Artemia* is associated with the accumulation of several chaperone proteins, including the small heat shock protein p26 [6, 7] and the diapause-specific ferritin homolog artemin [8], which are involved in embryo development, stress tolerance, and/or cyst discharge [9, 10]. However, the exact mechanism behind the instigation and termination of diapause is often unclear. It is perhaps this characteristic (i.e., cryptobiosis) that has facilitated *Artemia*’s transition from tributary to toy, and later, to television, and to space.

In 1957, dormant *Artemia* eggs were packaged into small, mail-order sachets within a self-contained tank and later commercialised under the name “Sea-Monkeys®” (originally “Instant Life”). In just a few weeks, the eggs would break their diapause, produce a few dozen adults, and introduce a generation of onlooking, budding aquarists to their new “instant pets” in the form of tiny, aquatic crustaceans. The exact details surrounding the Sea-Monkeys® themselves remain somewhat unclear. In promotional material, Sea-Monkeys® were described as not *A*. *salina* (a species often sold as feed) but rather an undisclosed “relative” (https://www.sea-monkeys.com/what-is-a-sea-monkey/) or “variety” (https://www.sea-monkeys.com/sea-monkey-set-up-instructions/sea-monkeys-handbook/), sometimes even a “hybrid”, referred to as *Artemia nyos* (https://www.sea-monkeys.com/what-is-a-sea-monkey/). However, it is unclear whether this claim is rooted in taxonomy or marketing, as *Artemia nyos* is not an accepted species name according to Zootaxa and sachets containing dormant eggs note “Assorted Mixed Salts & *Artemia [s]alina* Cysts/Eggs” as the only two main ingredients. Nevertheless, speciation in *Artemia* is complex, with morphologically similar species and ‘superspecies’ (*A*. *franciscana)* representing clusters of incipient species [1]. Misuse of the epithet *Artemia salina* is also common in historic literature [1]. Despite these claims remaining in instructional material, it is unclear whether such a hybrid is still in circulation given ongoing legal disputes. An affidavit from the current distributers of Sea-Monkeys® describes outsourcing animals from commercial *Artemia* producers due, in part, to their uncertain provenance [11].

**Fig 1:**
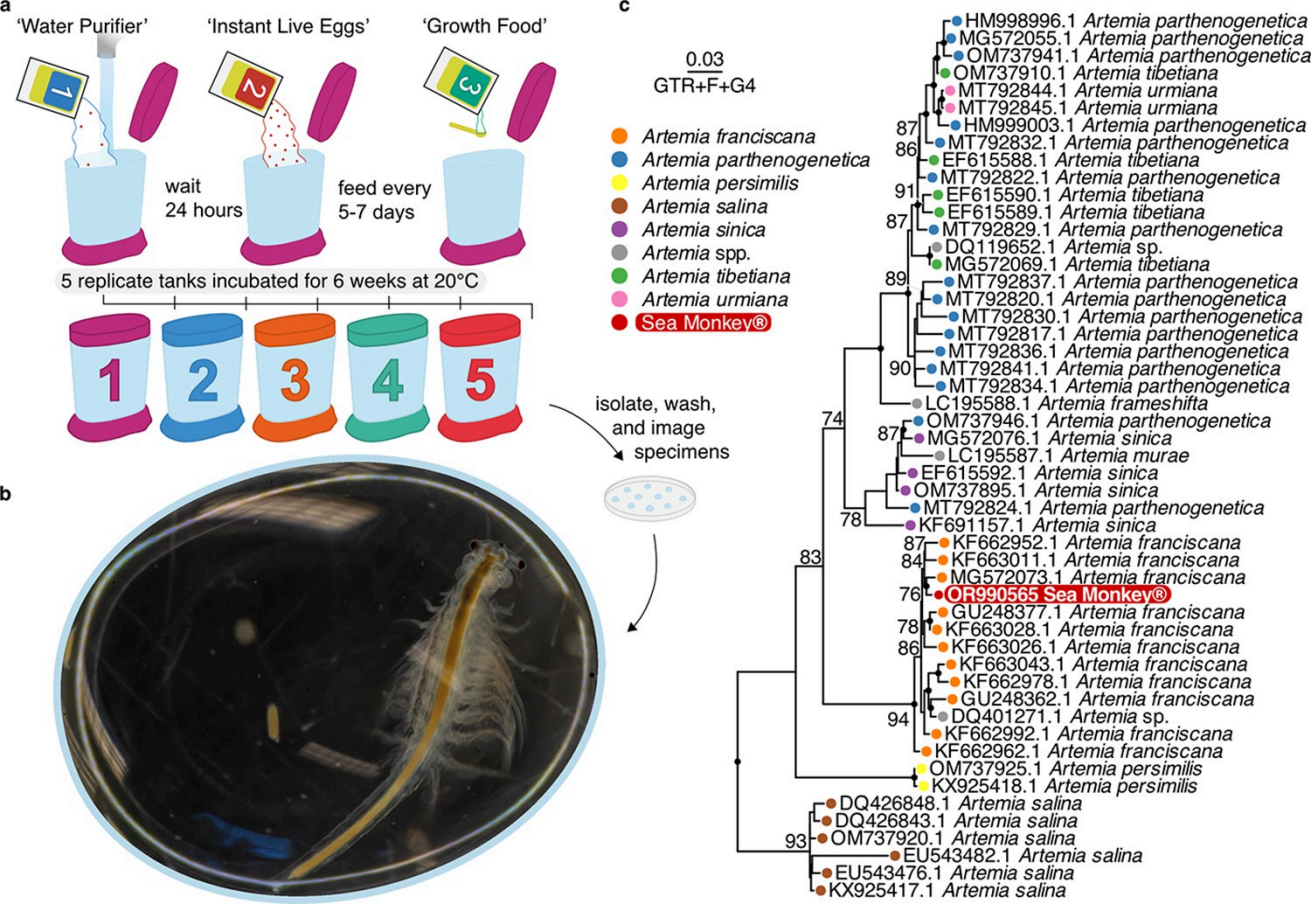
Sea-monkeys® sampling procedure, morphology, and phylogeny. **(A)** Schematic outlining the three-step process of Sea-Monkeys® culturing. **(B)** Sea-Monkey specimen photographed six weeks after the start of culturing. **(C)** Maximum-likelihood (ML) phylogenetic inference based on the COXI gene and a GTR+F+R4 substitution model. Black node labels indicate UltraFast bootstrap support 95% and above. Support values below 70% are omitted. Coloured tip labels show species identity of *Artemia* sequences deposited in Genbank. Outgroup not shown in plot.

Growing *Artemia* from Sea-Monkeys® kits is a three-step process involving three distinct sachets. Firstly, approx. 330ml of room temperature water is added to the container before adding the first sachet, the “Water Purifier”, which contains an assortment of salts (**Fig 1A**). Twenty-four hours later, the contents of the second sachet, “Instant Live Eggs”, can be added (**Fig 1A**), which supposedly will hatch instantaneously. After a further 5–7 days, the third and final sachet, “Growth Food”, can be added once a week using the provided scoop–the main ingredients of which are listed as “Assorted Mixed Salts & Organic Ground Vegetable Powder” (**Fig 1A**). On closer inspection, the first sachet also contains eggs, and the additional dye in the second sachet helps visualise those that have already hatched within the preceding 24 hours. If hatching is successful, nauplii undergo several moult cycles, progressing through multiple larval stages, and will eventually (somewhat) resemble the adult morphology characteristic of the brand (**Fig 1B**). A warning does note, however, that adding too much feed can lead to an overgrowth of bacteria which can “consume oxygen in the water, suffocating your pets”.

Crustacea, like other marine invertebrates, are known to live with microbiomes consisting of both prokaryotic [12, 13], eukaryotic [14–16], and viral communities [17, 18] and, likely owing to their commercial importance, *Artemia* spp. have been the subject of a small number of bacterial microbiome studies [19–22]. Therefore, one could presume that Sea-Monkeys® also harbour bacterial symbionts, despite the peculiar circumstance in which they find themselves. Bacterial microbiomes in aquatic invertebrates are highly influenced by the environment [13], with both location and habitat type exerting a significant and predictable effect on bacterial composition [13]. So, here, we investigate the influence of feed and tank-associated bacteria on Sea-Monkey microbiomes. Furthermore, we characterise the bacterial microbiome found within five separate Sea-Monkeys® kits grown over a 6-week period to determine whether there is any variation between distinct populations grown under the same conditions.

## Methods

### Ethics statement

This study did not involve any vertebrate animals or cephalopods.

### Sea-Monkeys® culturing and sampling

Sea-Monkeys® kits were obtained from Amazon. Prior to adding the contents of sachet 1, 330ml of distilled water were added to each tank. As noted, sachet 1 does contain *Artemia* eggs but, supposedly, its main purpose is to add salts to the tank water. Empty tanks (containing water and the contents of sachet 1) were left for 24 hours prior to the addition of Sea-Monkey eggs (sachet 2). Tanks containing Sea-Monkey eggs were incubated at 20°C on a 12-hour light cycle. Sachet 3 (the food source) was added after 5 days, and subsequently every seven days thereafter, using the kit supplied scoop (approx. 0.17 grams). After six weeks, all adults were removed from each tank using a disposable transfer pipette, separated into individual water droplets on a petri dish, and imaged (seven days after last feed). Each specimen was washed in sterile water (via successive transfers) and immediately immersed in lysis buffer from the QIAGEN QIAamp DNA Mini kit prior to mechanical tissue disruption and an overnight incubation with Proteinase K. After which, DNA was extracted following the kit manufacturer’s instructions, eluting in water.

After all animals were removed from the tank, the remaining water was removed using 50 ml syringes and filtered through Glass Fibre Filters (GF/F) in 3 batches of 100ml (comprising 3 distinct replicates). The GF/F filters were then cut into small pieces using sterile scalpels, added directly to the QIAGEN lysis buffer, and processed as above. One scoop of feed (sachet 3) from each Sea-Monkeys® kit was added directly to 5 separate replicates of DNA lysis buffer and also processed as above.

### Species identification using host marker genes

Host-derived, full-length 18S rRNA genes were amplified using the 18ScomF (5”- GCTTGTCTCAAAGATTAAGCCATGC-3”) and 18ScomR (5”- CACCTACGGAAACCTTGTTACGAC-3”) primers [23] and Phusion polymerase. Host-derived COXI genes were also amplified using LoboF1 (5”- KBTCHACAAAYCAYAARGAYATHGG-“3) and LoboR1 primers (5”- TAAACYTCWGGRTGWCCRAARAAYCA -3”) [24]. PCR reactions consisted of 12.5 μL of Phusion PCR Master Mix, 1.25 μL of each primer, 7.5 μL of water and 2.5 μL of DNA template. Thermal cycler parameters for both 18S and COXI genes consisted of an initial denaturation at 98°C for 30s, followed by 30 cycles of 98°C for 10s, 65°C/54°C for 30s (respectively) and 72°C for 30s, and a final extension at 72°C for 5mins.

PCR products were cleaned via gel electrophoresis, extracted with the NEB Monarch Gel Extraction kit, and sequenced using the ABI Big Dye 3.1 Cycle Sequencing kit. Reads were trimmed according to accompanying electropherograms, merged to create consensus sequences, and aligned against reference sequences obtained from GenBank using the EINSI algorithm in mafft (v. 7.490) [25]. All sequences (excluding the outgroup) were clustered at 98% with cd-hit-est (v.4.8.1) [26] prior to aligning to aid visualisation. The alignment was then lightly masked with trimal (v.1.4.rev22) using a gap threshold of 0.3 and similarity threshold of 0.001 [27]. A Maximum Likelihood (ML) phylogeny was inferred using IQTREE (v.1.6.12) and the GTR+F+R4 substitution model (chosen using -mset GTR) [28]. The final tree was then plotted In R (v.4.3.0) [29] using the ggtree package (v.3.8.2) [30].

### Microbiome library preparation and sequencing

Amplicon libraries were prepared and sequenced by the Integrated Microbiome Resource (IMR), Halifax, Canada, according to their standardised protocol (https://imr.bio/protocols.html). The DNA template was diluted using 1:1 and 1:10 ratios and PCR-amplified using high-fidelity Phusion Plus polymerase and “fusion primers” containing Illumina adaptors and indices. The prokaryote-specific primers 515FB (5”- GTGYCAGCMGCCGCGGTAA-3”) and 806RB (5”- GGACTACNVGGGTWTCTAAT-3”) were used to amplify the V4 region of the 16S rRNA SSU gene [31]. Replicate dilutions were then pooled and the amplicons were cleaned using the Charm Biotech Just-a-Plate 96-well Normalization Kit. All samples were subsequently pooled, quantified with a fluorometer, and sequenced on an Illumina Miseq using paired-end 300bp reads.

### Microbiome analysis and statistics

Primers were first removed from raw reads using cutadapt (v.3.4) [32] before ASV processing using the DADA2 pipeline (v.1.28.0) [33] in R (v.4.3.0) [29]. Reads were filtered and trimmed according to their quality profiles and the following parameters: maximum “expected error” (maxEE) values of 2 (for both forward and reverse reads), truncate reads when the quality score reaches 2 (trunQ), and remove all reads containing Ns. The parametric error model was generated using the default number of bases (1e^8^) and the sample inference algorithm was subsequently applied using “pseudo” pooling, which allows the detection of singletons by sharing information between independently processed samples. Reads were then merged, and chimeras were removed before assigning taxonomy using the naïve Bayesian classifier method [34] (default minimum bootstrap support value of 50%) and the SILVA database (v.138.1). The resulting ASV count table and corresponding taxonomic assignments are combined with sample-specific metadata using the phyloseq package (v.1.44.0) [35].

**Fig 2:**
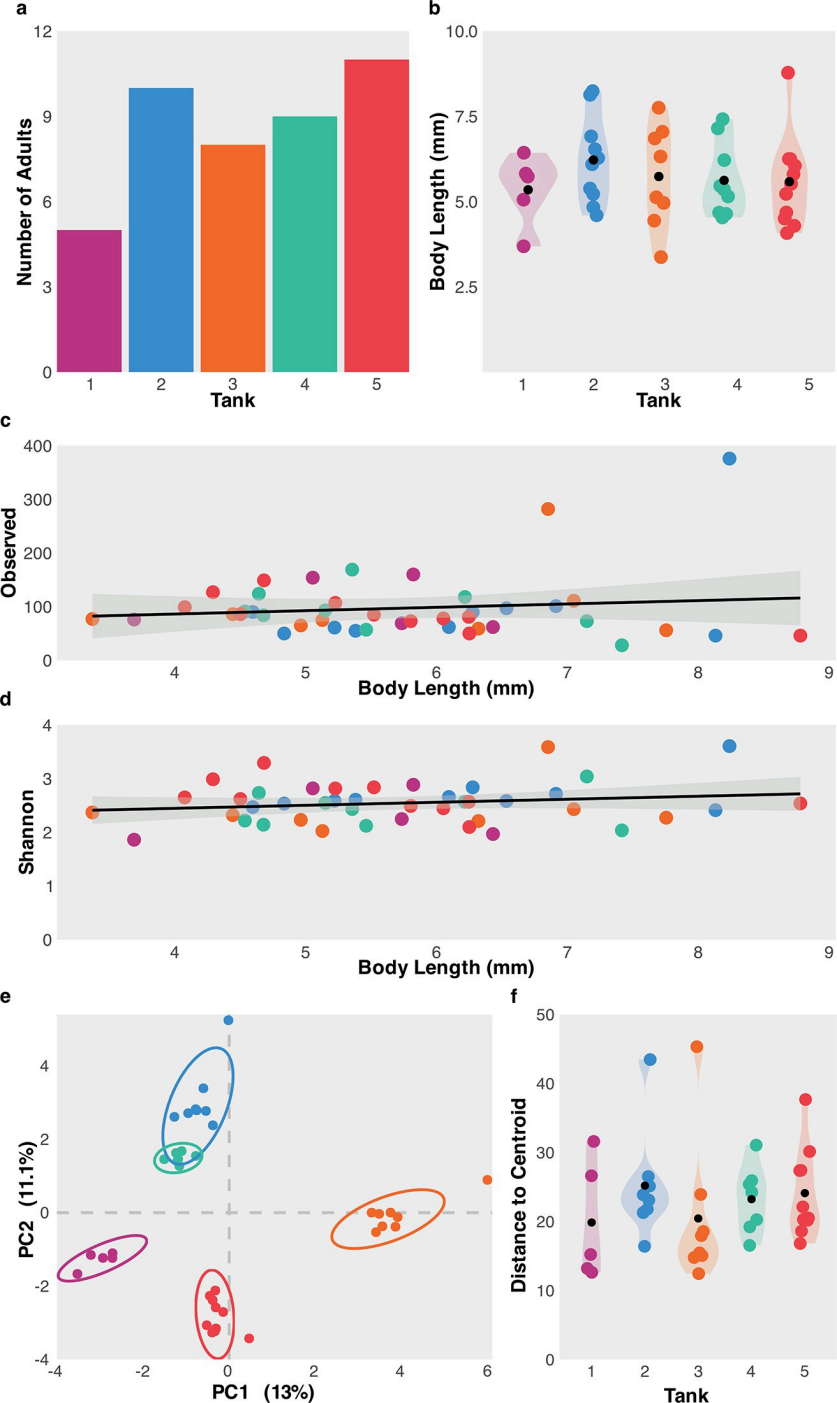
Variation between individual tanks. **(A)** Number of adults in each tank at the time of sampling. **(B)** Distribution of body length (mm) per tank. **(C)** Correlation between body length (mm) and observed ASV richness. **(D)** Correlation between body length (mm) and observed Shannon Diversity Index. Regression plotted using “lm” method and formula ‘y ~ x’. **(E)** PCA of Sea-Monkeys® microbiomes using Atchison distance. **(F)** Distance to group centroids in ordination (variance within group) per tank. Individual points coloured according to tank identity. Black points in violin plots show average values.

Contaminant sequences were identified and removed using the SCRuB package (v.1.0.0) [36], incorporating 96-well plate location of each sample. ASVs assigned to Eukaryotes were also removed for all analyses excluding the depiction of plastid sequences in feed (**Fig 2C**). Samples that contained fewer than 500 reads were also removed. The final dataset for each tank contained (on average and respectively from Tank 1:5) 5912, 3327, 4260, 5870, and 3328 reads for animals; 684, 1097, 985, 951, and 906 reads for feed; and 54536, 62008, 51468, 65201, 75600 for water. Shannon’s Diversity Index and observed ASV richness was calculated (for animal-associated microbiomes) using the estimate_richness function from the phyloseq package. Correlations between body length and alpha diversity were analysed using the Pearson method and cor.test function from the core stats package (v.4.3.0) [33].

Prior to Principal Components Analyses (PCA) using the phyloseq ordinate() function [35], ASVs with a minimum relative abundance above 0.005% across all samples were removed and ASV counts were normalised into centred-log ratios using the microbiome package (v.1.22.0) [37]. Permutational Multivariate Analysis of Variance (PERMANOVA) and multivariate homogeneity of groups dispersions (betadisper) were tested with the adonis2 and betadisper functions (respectively) of the vegan package (v.2.6.4) [38] using 1000 permutations. Pairwise Tukey tests using the ‘Honest Significant Differences’ method was computed with the TukeyHSD function of the core stats package [33].

To quantify and visualise shared ASVs between sample types, individual samples from each tank were merged in phyloseq to produce a representative sample from each group (Feed_1, Adult_1, Water_1, Feed_2 etc.). Shared ASVs between sample types were visualised using the ggalluvial (v.0.12.5) [39] and ggvenn (v.0.1.10) [40] packages, with only those with a relative abundance greater than 0.01% included in the former (for clarity). The randomForest function from the randomForest package (v.4.7.1.1) [41] was used to predict sample type and source tank using 50 trees. Unless stated, all plots were generated with ggplot2 (v.3.4.3) [42] and exported to Adobe Illustrator final edits and rearrangement.

## Results and discussion

### Sea-Monkeys® likely belong to the *A*. *franciscana* superspecies

Full-length rRNA 18S (OR989981) and mitochondrial COXI (OR990565) gene sequences obtained from Sea-Monkey specimens share 99.88% and 99.55% identity to reference sequences annotated as *A*. *salina* and *A*. *franciscana*, respectively. Notably, the Sea-Monkey 18S sequence is also 99.70% identical to that of *A*. *franciscana*. Unlike the COXI gene marker, there are very few *Artemia* 18S sequences deposited in GenBank (6 accessions in total) and this likely confounds its interpretation. Furthermore, nucleolar dominance (an epigenetic phenomenon where one progenitor’s rRNA genes are silenced [43]) might result in a high identity to a single species, further obfuscating molecular identification of a potential hybrid using ribosomal gene markers. There is, however, an extensive number of *Artemia* COXI accessions and maximum-likelihood phylogenetic inference shows the Sea-Monkey COXI sequence clustering firmly within a clade of reference sequences annotated as *A*. *franciscana* (**Fig 1C**).

*Artemia franciscana* and *A*. *salina* are commonly used in aquaculture. *Artemia franciscana* is, however, the dominant species in the ‘New World’ and several strains have since been introduced elsewhere, resulting in reproductive isolation and its classification as a ‘superspecies’. Cross-fertility tests within this species complex show mixed results [44] and successful hybrids show varying traits; hybrids of *A*. *franciscana* and *A*. *persimilis*, for example, showed a greater percentage of encysted offspring [44]. These results do not clearly distinguish if Sea-Monkeys® are a hybrid (if indeed such a hybrid is still used): the phylogenetic placement of the maternally inherited COXI sequence suggests that at least the maternal line likely falls withing the *A*. *franciscana* species complex.

### My Sea-Monkeys® are better than your Sea-Monkeys®

Despite shared culture conditions, the number of adults at the time of sampling varied between tanks: ranging from 5 to 11 individuals (**Fig 2A**). This variation is likely explained by serval factors, including the numbers of eggs added to each tank, differential rates of hatching between tanks, and variable mortality of hatchlings thereafter. There was no significance difference in body size between tanks (ANOVA, *p* value = 0.635) with an overall average body length of 5.74 mm (**Fig 2B**), however adults ranged from 3.4 mm to 8.8 mm.

One might expect microbiome diversity to increase as a function of animal size simply due to a greater surface area for colonisation [45]. Indeed, gut volume is a major driver of bacterial microbiome diversity in vertebrates [46]. However, observed ASV richness (**Fig 2C**) and Shannon’s diversity index (**Fig 2D**) of the Sea-Monkey microbiome did not significantly correlate with an increase in body length of the host (PEARSON’S: Shannon, cor = 0.181, *p* value = 0.243; Observed ASVs, cor = 0.126, *p* value = 0.421).

Individual tanks have pronounced effects on microbiome composition, with Principal Coordinates Analysis (PCA) based on normalised read counts (Atchison distance) showing individuals from different tanks having significantly dissimilar microbiomes (PERMANOVA, *p* value < 0.001; **Fig 2E**), despite similar variances within tanks (BETADISPER, p-value = 0.671; **Fig 2F**). For the most part, these tanks effects are also predictable, with random forest models using ASV counts to predict tank identity showing an overall out-of-bag (OOB) error rate of 10.34%.

### General trends in Sea-Monkey microbiomes

**Fig 3:**
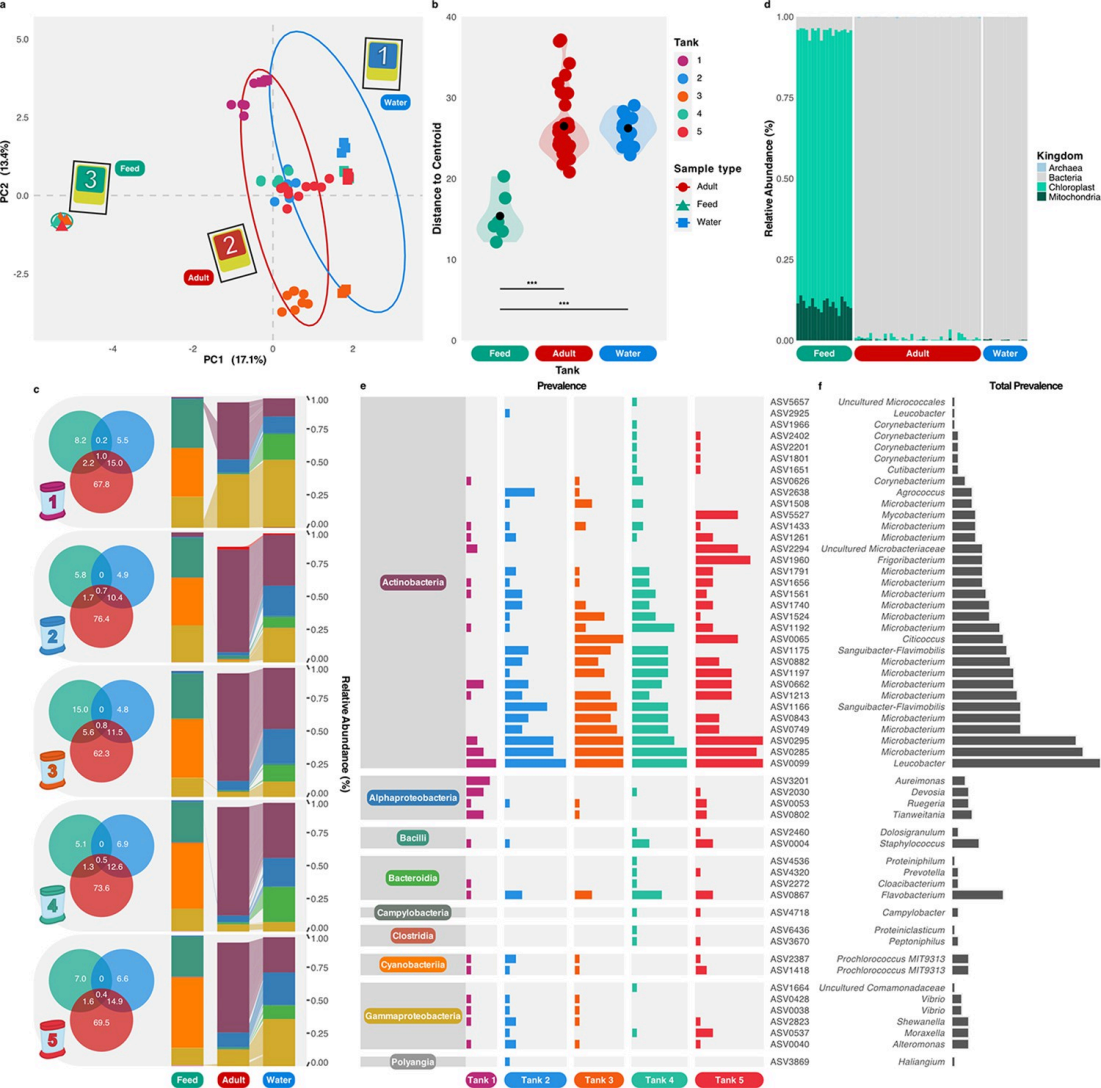
Fine-scale taxonomic differences between Sea-Monkey Microbiomes. **(A)** PCA of Sea-Monkey® adult, feed and tank water microbiomes using Atchison distance. **(B)** Distance to group centroids in ordination (variance within group) for feed, adult, and water microbiomes. Individual points coloured according to tank identity. Black points in violin plots show average values. **(C)** Proportion of all tank-associated ASVs shared between sample groups depicted by Venn diagram (values rounded to 1 decimal point). Average relative abundance profiles of taxa coloured according to **E** are shown in the accompanying bar chart. Shared ASVs between Adult-Feed and Adult-Water are shown as links between stacked bars. Unlike Venn diagrams, bar charts only include ASVs above 0.01% relative abundance to aid visualisation of taxa. **(D)** Relative abundance of broad taxonomic assignments showing dominance of eukaryotes in feed samples assigned to chloroplast or mitochondria. **(E)** Relative prevalence of adult-only ASVs above 0.01% relative abundance (separated according to bacterial Class) in each tank. **(F)** Total prevalence of adult-only ASVs.

Sea-Monkey microbiome composition across all tanks is significantly different from that of the water and the feed (PERMANOVA, *p* value < 0.001; **Fig 3A**). However, there is a much smaller variance in feed microbiomes compared to the adult and water (BETADISPER, p-value = 0.001; **Fig 3B**), and this can impact ordination statistics. Indeed, pairwise Tukey comparisons confirm that microbiome dispersion is not significantly different when comparing adult and water (p-value = 0.967). Random forest models showed ASV counts were also reliable predictors of adult, feed, or tank microbiome origin, and did so with a much lower OOB error rate of just 1.72%.

A relatively small proportion of the total tank-associated ASVs were shared between Sea-Monkeys® and the water (10.4%, tank 2, blue; to 15% tank 1, violet; **Fig 3C**) but these ASVs dominated Sea-Monkey microbiomes in terms of relative abundance i.e., adult specimens contained a greater number of low-abundant taxa absent in other sample groups (**Fig 3C**). On average, 2.2% ± 1.8% (SD) of tank-associated ASVs were shared between animals and the feed (**Fig 3C**). However, the majority of sequences identified in the feed were derived from plastidial sequences (**Fig 3D**). These were also detected at low relative abundance in animals but removed from all analyses to prevent an inaccurate view of protist diversity (as not all protists bear plastids). The dominant plastid sequence is 100% identical to both *Trigonella foenum-graecum* (fenugreek) and several *Medicago* spp. (alfalfa), common plants that could well be used as the supplied food source.

Actinobacteria was the most abundant bacterial phyla in all but one of the tanks, which was instead dominated by Gammaproteobacteria (**Fig 3C**). Micrococcales were by far the most common Actinobacteria taxa, followed by Rhizobiales. In previous studies, high proportions of Actinobacteria were found in the microbiome of *Artemia* experimentally fed either polyethylene or polystyrene microplastics [22]. Microbiomes from control animals instead harboured a Proteobacteria-dominated community. Firmicutes and Proteobacteria were also noted to dominate wild-caught *Artemia* [19]. In fact, Proteobacteria are often the most substantial proportion of bacteria taxa in penaeid shrimp microbiomes [47]. However, rather than an effect induced by the plastics in the tank, an Actinobacteria-heavy community might reflect the vegetable powder diet, as many marine species produce enzymes capable of degrading common plant polysaccharides like cellulose and xylan [48]. Thus, despite a negligible overlap between animal- and feed-associated microbes, nutrient composition may well serve as a selection pressure driving composition in *Artemia* microbiomes.

### Could host-specific bacterial variants protect against desiccation?

Most animal-specific ASVs were also Actinobacteria but these tend to represent low relative abundances. *Leucobacter* (ASV0099) is one of the most prevalent animal-only genera (**Fig 3E and 3F**). The corresponding ASV sequence is identical to several species deposited in GenBank so its exact identity is unknown. However, *Leucobacter* are found in diverse environments and in associated with many hosts [49]. *Leucobacter* are also remarkable nematopathogenic bacteria and known for causing star-shaped aggregates of nematodes known as “worm-stars” [50]. The role of *Leucobacter* in Sea-Monkeys® is unclear. No obvious gross pathologies were observed and *Leucobacter* ASVs were in all tanks, regardless of the number of adults at the time of sampling.

Several *Microbacterium* ASVs were also restricted to animal microbiomes (**Fig 3E**). However, unlike *Leucobacter*, the genus itself was also found in the surrounding water. *Microbacterium*, being a widespread genus in soil and aquatic environments, has been reported as a contaminant in laboratory reagents but it is not out of place in this setting. *Microbacterium* is also reported as a common genus in other, including wild, *Artemia* [19, 22].

Notably, both *Leucobacter* and *Microbacterium* have previously shown a high tolerance to desiccation [51] and both can protect plants from draughts [52, 53]. The *Leucobacter sp*. 4J7B1 genome includes several genes known for their role in glycerol metabolism, a well-known osmoprotectant [54], whereas the *Microbacterium* sp. strain 3JI genome encodes antioxidants with important roles in desiccation tolerance [55]. Both genera (but particularly *Microbacterium*) have been associated with trehalose production, hypothesised to facilitate their survival (as well as that of their plant host) during desiccation [53]. Artemia cysts also contain large amounts of trehalose, while non-dormant embryos do not [56]. The water replacement hypothesis suggests that trehalose might replace water molecules in membranes as cells dehydrate [57]. Notably, trehalose has also been implicated in other cryptobiotic animals [56]. Coincidentally, glycerol also accumulates in *Artemia* cysts but is thought to contribute to the rupturing of the shell by changing internal osmotic pressure [58]. The detection of both *Leucobacter* and *Microbactrium* in *Artemia* could simply be a result of desiccation as a clear selective pressure (although the former was absence in feed and water samples). Alternatively, as these bacteria are capable of conferring such a benefit to plant hosts, perhaps, a potential bacterial symbiont involved in *Artemia’s* vital tolerance to desiccation should not be dismissed at this stage.

Notably, one of the few papers referencing “Sea-Monkey” describes a case of a persistent *Mycobacterium marinum* infection potentially derived from the animals themselves [59]; shrimp (among other animals) are known vectors of the bacterium, which is a common cause of cutaneous mycobacterial infections in aquarists. *Mycobacterium* was indeed found in Sea-Monkey microbiomes from three tanks; but only once occurred with a relative abundance above 1% (**Fig 3E**).

## Conclusion

Sea-Monkeys® harbour bacterial microbiomes which show similar taxonomic compositions but differ at the ASV level. Adult microbiomes are more similar to the microbial communities of the tank water, but several adult-only bacterial variants were detectable. The source of these ASVs is unclear, given their absence in both feed and water. One possibility is that some host-specific taxa are vertically transmitted and themselves remain dormant within the dormant egg. We were unable to generate libraries from isolated eggs to test this hypothesis. The presence of bacteria known to confer tolerance to desiccation raises interesting question about the role of symbiosis in this remarkable reproduction strategy. However, this would require more comprehensive metagenomic sequencing. Multi-generational studies are needed to determine whether bacteria like *Leucobacter* are indeed important symbionts for anhydrobiotic animals like *Artemia* and whether they are transmitted from parent to offspring. Given their role as animal feed in aquaculture, a greater awareness of any such microbes involved in *Artemia* development would be a useful tool for improving future production. As discrete experimental units, Sea-Monkeys® could well serve as a useful resource for testing such theories in the future but arguably their greatest potential, facilitated by their familiarity, accessibility, and simplicity, is as a tool for science communication and education.
